# Statin Use and Risk of Prostate Cancer: A Meta-Analysis of Observational Studies

**DOI:** 10.1371/journal.pone.0046691

**Published:** 2012-10-01

**Authors:** Dipika Bansal, Krishna Undela, Sanjay D'Cruz, Fabrizio Schifano

**Affiliations:** 1 Department of Pharmacy Practice, National Institute of Pharmaceutical Education and Research, S. A. S. Nagar (Mohali), Punjab, India; 2 Department of General Medicine, Government Medical College and Hospital, Chandigarh, India; 3 School of Pharmacy, University of Hertfordshire, Hatfield, Herts, United Kingdom; Copenhagen University Hospital Gentofte, Denmark

## Abstract

**Background:**

Emerging evidence suggests that statins may decrease the risk of cancers. However, available evidence on prostate cancer (PCa) is conflicting. We therefore examined the association between statin use and risk of PCa by conducting a detailed meta-analysis of all observational studies published regarding this subject.

**Methods:**

Literature search in PubMed database was undertaken through February 2012 looking for observational studies evaluating the association between statin use and risk of PCa. Before meta-analysis, the studies were evaluated for publication bias and heterogeneity. Pooled relative risk (RR) estimates and 95% confidence intervals (CIs) were calculated using random-effects model (DerSimonian and Laird method). Subgroup analyses, sensitivity analysis and cumulative meta-analysis were also performed.

**Results:**

A total of 27 (15 cohort and 12 case-control) studies contributed to the analysis. There was heterogeneity among the studies but no publication bias. Statin use significantly reduced the risk of both total PCa by 7% (RR 0.93, 95% CI 0.87–0.99, p* = *0.03) and clinically important advanced PCa by 20% (RR 0.80, 95% CI 0.70–0.90, p<0.001). Long-term statin use did not significantly affect the risk of total PCa (RR 0.94, 95% CI 0.84–1.05, p = 0.31). Stratification by study design did not substantially influence the RR. Furthermore, sensitivity analysis confirmed the stability of results. Cumulative meta-analysis showed a change in trend of reporting risk from positive to negative in statin users between 1993 and 2011.

**Conclusions:**

Our meta-analysis provides evidence supporting the hypothesis that statins reduce the risk of both total PCa and clinically important advanced PCa. Further research is needed to confirm these findings and to identify the underlying biological mechanisms.

## Introduction

Prostate cancer (PCa) is the sixth leading cause of cancer death in males worldwide [1]. The developed countries carry most of the disease burden, accounting for nearly three quarters (72%) of the total in 2008 [2]. It is the second leading cause of cancer death in American men, after lung cancer [3].

Statins (3-hydroxy-3-methyl glutaryl-coenzyme A reductase inhibitors), a group of cholesterol lowering drugs, have shown PCa growth inhibiting potential both in animal [4] and clinical studies [5]–[7]. However, evidence on statins effect on overall PCa risk has been more controversial, with some studies having not identified any effect [8]–[10], others having described an increased overall PCa risk [11]–[14], whilst remaining studies having reported reduced overall risk [15]–[17]. Some randomized clinical trials (RCTs) on statin use in coronary heart disease [18], [19], report non-significant decreased incidence of PCa among statin users compared to non users, but most of the results were ambiguous because of inadequate power. Currently there is only one on-going clinical trial (simvastatin vs. placebo) which examines the biologic effects of statins on prostate cancer in humans [20].

This issue was discussed in previously conducted meta-analyses' [21]–[23] which have analyzed statins chemopreventative effect in overall cancers and site-specific cancers. No significant association was found between statin use and total PCa risk in these studies. A recent meta-analysis done by Bonovas *et al*. [24], focussing on PCa risk in statin users, included 6 RCTs and 13 observational studies published between 1993 and 2007, and reported no association. In contrast, they concluded that there was a negative association between statin use and advanced PCa risk. However, 17 more studies [5]–[7], [25]–[38] evaluating the association between statins use and risk of PCa were published after 2007. In the present meta-analysis, we examined statin use in relation to total PCa and also clinically important advanced PCa, taking into account most recent studies.

## Materials and Methods

### Literature Search

Two authors independently performed the literature search by using PubMed Database up to February 2012. Search terms include: “statin(s)” or “HMG-CoA reductase inhibitor(s)” or “lipid-lowering agent(s)” or “atorvastatin” or “cerivastatin” or “fluvastatin” or “lovastatin” or “mevastatin” or “pravastatin” or “rivastatin” or “rosuvastatin” or “simvastatin” and “cancer(s)” or “neoplasm(s)” or “malignancy(ies)” with limits; Humans and English. The titles and abstracts of the resulting articles were examined to exclude irrelevant studies. The full texts of remaining articles were read to extract information on the topic of interest. Bibliographies and citation sections of retrieved articles were also reviewed for additional pertinent studies.

### Inclusion and exclusion criteria

The studies considered in this meta-analysis were all observational (cohort or case-control) studies that evaluated exposure to statins and risk of PCa. Any discrepancies were addressed by a joint re-evaluation of the original article. Articles were excluded if they were reviews, letters to the editor without original data, editorials and case reports. When there were multiple publications from the same population, only data from the most recent report were included in the meta-analysis and remaining were excluded [39]–[42].

### Data extraction

Two authors independently reviewed the primary studies to assess the appropriateness for inclusion in the present meta-analysis and data were extracted. The following information was assayed from each study: *(i)* first author's last name, year of publication, and country of the population studied; *(ii)* study design; *(iii)* number of male subjects and number of PCa cases; *(iv)* relative risk (RR) estimates and 95% confidence intervals (CIs); *(v)* definitions of statin exposure, long-term statin use and advanced PCa; *(vi)* PCa assessment; and *(vii)* control for confounding factors by matching or adjustments, if applicable. We extracted the RR estimates that reflected the greatest degree of control for potential confounders.

### Quality assessment

The quality of each study was assessed independently by two authors by using the Newcastle-Ottawa Scale (NOS) [43]. The NOS consists of three parameters of quality: selection, comparability, and outcome (cohort studies) or exposure (case-control studies). The NOS assigns a maximum of four points for selection, two points for comparability, and three points for exposure/outcome. Therefore, nine points reflects the highest quality. Any discrepancies were addressed by a joint revaluation of the original article with a third author.

### Data synthesis and analysis

Because the risk of PCa is low, the RR in prospective cohort studies mathematically approximates the odds ratio [44], therefore permitting the combination of cohort and case-control studies. Publication bias was assessed using Begg and Mazumdar adjusted rank correlation test and Egger regression asymmetry test [45], [46]. To assess the heterogeneity among studies, we used the Cochran *Q* and *I^2^* statistics; for the *Q* statistic, a p value<0.10 was considered statistically significant for heterogeneity; for *I^2^*, a value >50% is considered a measure of heterogeneity [47]. The primary measure was pooled RR of PCa from individual studies, calculated using the random-effects model (DerSimonian and Laird method), which accounts for heterogeneity among studies. Tests for interaction using summery estimates were performed, using the method described by Altman and Bland [48]. All analyses were performed using STATA version 11.0 (StataCorp, College Station, TX). All statistical tests were two-sided and p<0.05 was considered statistically significant, except otherwise specified.

The primary outcome in this meta-analysis was reported as RR with 95% CI of developing PCa in statin users. To assess any link between *(i)* long-term statin use and total PCa, *(ii)* statin use and, specifically, advanced PCa, we used the available data from studies which reported RR estimates for these particular associations.

Subgroup analyses were performed according to *(i)* study design (cohort and case-control), *(ii)* adjustment for prostate specific antigen (PSA) testing *(iii)* adjustment for body mass index (BMI) and/or adverse life style (ALS), and *(iv)* studies before and after Bonovas *et al*., analysis [24], to examine the impact of these factors on the association. To evaluate the stability of our results, we also performed a one-way sensitivity analysis. The scope of this analysis was to evaluate the influence of individual studies by estimating the average RR in the absence of each study. Cumulative meta-analysis was also performed to identify the change in trend of reporting risk over time. The present work was performed as per the guidelines proposed by the Meta-analysis of Observational Studies in Epidemiology group [49] and Preferred Reporting Items for Systematic Reviews and Meta-Analyses (PRISMA) (**Checklist S1**).

## Results

### Search results

**Figure 1:**
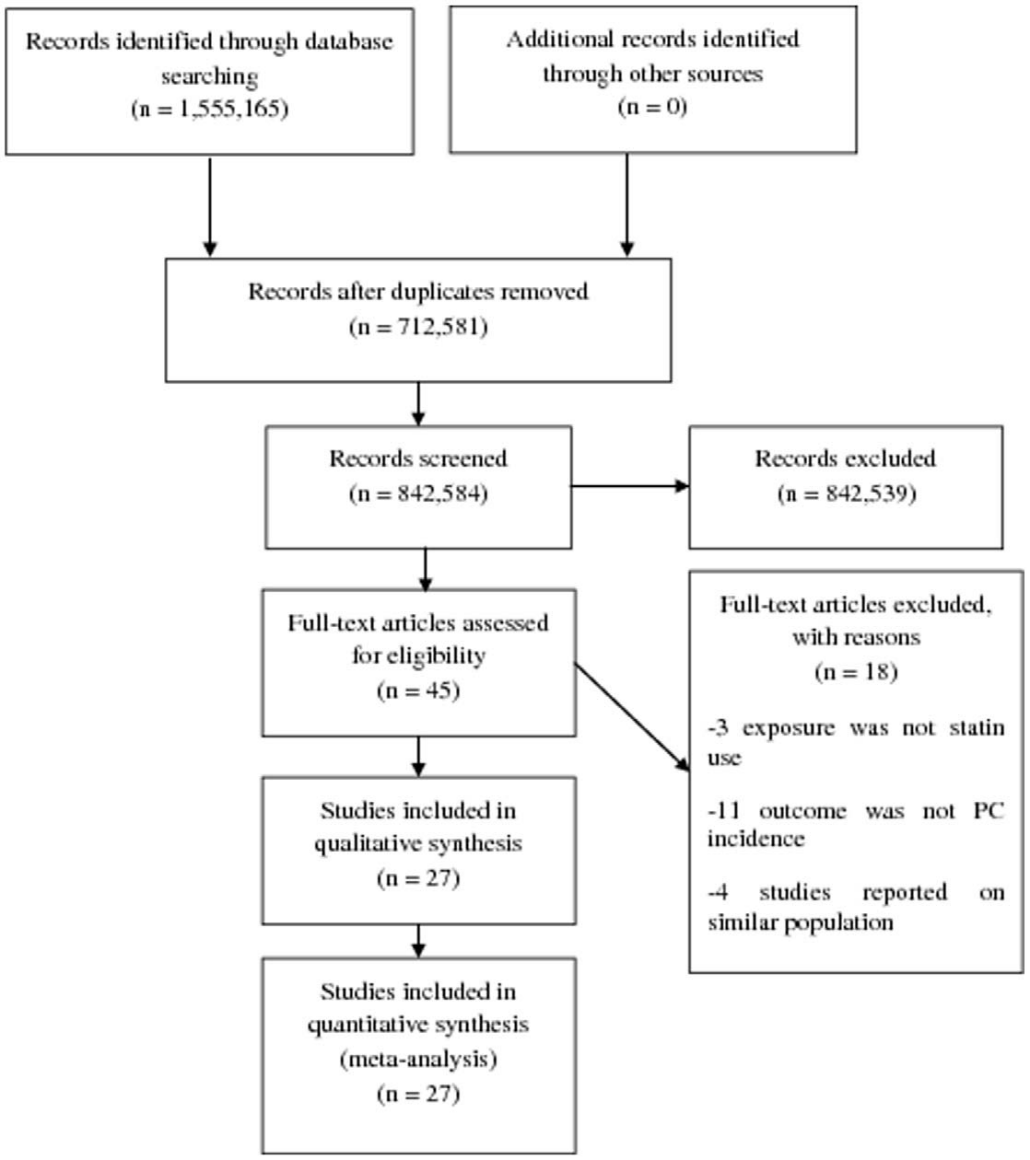
Flowchart representing the selection process.

A total of 1,555,165 articles were identified during the initial search (**figure 1**). After reviewing the titles and abstracts of these articles, 1,555,120 were found to be ineligible as they were reviews, editorials, case reports and others did not met the inclusion criteria. After detailed evaluation of the remaining 45 full-text articles, 18 were excluded for reasons described in **figure 1**.

### Study characteristics

Twenty seven relevant studies were identified, including 15 cohort and 12 case-control studies involving a total of 1,893,571 male subjects including 56,847 PCa cases. Participants were followed-up for 2 to 17 years and the studies have been published between 1993 and 2011.

Fifteen cohort studies of statin use and risk of PCa were published between 1993 and 2011 included 1,812,005 participants, followed-up for 2 to 17 years, reporting a total of 5,770 incident PCa cases among 518,278 statin users, whereas 10,375 incidents of PCa cases among 1,258,019 non-statin users. Five studies reported a negative association between statin use and risk of total PCa [5], [6], [25], [26], [28]. All studies assessed PCa diagnosis through cancer registry, except for 3 [6], [10], [26] which assessed diagnosis through medical records. Of the total fifteen cohort studies, seven were conducted in United States (US) [8], [10], [25], [26], [28], [34], [38], six in Europe [5], [6], [9], [30], [35], [36], one in both US and Europe [11], and one in Asia [13].

Twelve case-control studies have been published between 2000 and 2011. These studies included 81,566 participants, followed-up for 3 to 13 years, reporting a total of 3,550 statin users among 31,862 PCa cases and 3,325 statin users among 40,872 controls. Six studies reported a negative association between statin use and risk of total PCa [7], [15]–[17], [27], [29]. Statin use was ascertained by review of medical records in 8 studies [7], [12], [14]–[17], [29], [32] and by mailed questionnaires in 4 studies [27], [31], [33], [37]. Of them, eight studies were conducted in US [7], [15], [17], [27], [29], [31], [33], [37], three in Europe [12], [14], [16], and one in Asia [32].

All studies evaluated exposure specifically to statins and the risk of PCa except for 2 studies [14], [38] that examined the use of all cholesterol-lowering drugs like fibrates and bile acid-binding resins along with statins. All studies were controlled for potential confounding factors (at least for age) by matching or adjustment. The characteristics of the selected studies are presented in **table 1**.

**Table 1:** Studies included in the meta-analysis.

Author, Year* (Country)†	Study period (years)	All male subjects	PCa cases	Description of exposure∥	Definition of statin use¶	Number of variables adjusted#
Lovastatin study groups, 1993 (U.S., Canada & Finland) [11] ‡	NR	504	5	a	A	1
Blais *et al*., 2000 (Canada) [15] §	6 (1988–1994)	858	78	b	NR	1, 27, 31, 33, 34
Graaf *et al*., 2004 (Netherlands) [16] §	3 (1995–1998)	9,785	186	c	NR	1, 3, 5, 11–13, 27, 29–31
Kaye and Jick, 2004 (U.K.) [12] §	12 (1990–2002)	8,020	569	d	B	1, 4, 19, 32
Friis *et al*., 2005 (Denmark) [9] ‡	13 (1989–2002)	168,133	1407	e	C	1, 5, 28, 29
Shannon *et al*., 2005 (U.S.) [17] §	7 (1997–2004)	302	100	e	C	1–5, 25, 27
Platz *et al*., 2006 (U.S.) [10] ‡	12 (1990–2002)	34,989	2,579	d	A	1, 3, 4, 8, 10, 19–26
Sato *et al*., 2006 (Japan) [13] ‡	14 (1991–2005)	215	2	f	A	1
Flick *et al*., 2007 (U.S.) [8] ‡	2 (2002–2004)	69,047	888	g	B	1–3
Murtola *et al*., 2007 (Finland) [14] §	7 (1995–2002)	49,446	24,723	g	C	1, 11–17
Boudreau *et al*., 2008 (U.S.) [25] ‡	2 (1990–2005)	83,372	2,532	g	C	1, 3, 5, 7, 27
Friedman *et al*., 2008 (U.S.) [34] ‡	9 (1994–2003)	NR	1,706	e	B	35
Smeeth *et al*., 2008 (U.K.) [35] ‡	11 (1995–2006)	364,675	3,525	d	B	1, 3, 9, 11–14, 27, 28, 35–38
Agalliu *et al*., 2008 (U.S.) [37] §	13 (2002–2005)	1,943	1,001	d	A	1, 2, 4, 8, 19
Breau *et al*., 2010 (U.S.) [26] ‡	17 (1990–2007)	2,447	224	d	A	1, 3, 5, 9, 39–41
Haukka *et al*., 2010 (Finland) [30] ‡	9 (1996–2005)	10,928	1051	d	C	1, 42
Hippisley *et al*., 2010 (England & Wales) [36] ‡	6 (2002–2008)	990,495	7,129	d	B	NR
Murtola *et al*., 2010 (Finland) [5] ‡	8 (1996–2004)	23,208	1,594	d	C	1, 8, 10, 12–17, 24, 35
Coogan *et al*., 2010 (U.S.) [31] §	6 (1992–2008)	3,374	1,367	e	A	2, 4–6, 18, 19, 32, 43, 44
Loeb *et al*., 2010 (U.S.) [27] §	6 (2003–2009)	1,351	1,351	e	B	45
Farwell *et al*., 2011 (England) [6] ‡	10 (1997–2007)	55,875	546	h	B	1, 3, 7–9, 18, 19, 39, 46–52
Tan *et al*., 2011 (Ohio) [28] ‡	10 (2000–2010)	4,204	1,797	g	B	1, 2, 4, 53, 54
Jacobs *et al*., 2011 (U.S.) [38] ‡	10 (1997–2007)	3,913	NR	i	A	1–10, 18
Chang *et al*., 2011 (Taiwan) [32] §	3 (2005–2008)	1,940	388	g	C	3, 5, 9, 27, 32, 39, 55, 56
Fowke *et al*., 2011 (U.S.) [33] §	8 (2002–2010)	2,148	1029	g	A	1–4, 9, 8–10, 24, 45, 54, 55
Mondul *et al*., 2011 (Maryland) [29] §	13 (1993–2006)	2,399	683	d	A	1, 2, 4, 10, 13, 19, 24
Marcella *et al*., 2011 (New Jersey) [7] §	3 (1997–2000)	767	387	g	B	1, 2, 4, 6, 13, 57, 58

PCa, Prostate cancer; NR, Not reported.

* Publication year;

† Country of study conducted;

‡ Cohort studies;

§ Case-control studies.

∥ a, systematic use of lovastatin *vs.* SEER data; b, any use of statin *vs.* use of bile acid-binding resins; c, use of statins *vs*. no use of statins; d, current use of statins *vs*. no current use of statins; e, any use of statins *vs.* no use of statins; f, systematic use of statins *vs.* general population; g, ever use of statins *vs.* no use of statins; h, use of statins *vs*. use of anti-hypertensives; i, current use of cholesterol-lowering drugs *vs.* never use of cholesterol-lowering drugs.

¶ A, self-reported; B, medical records; C, prescription database.

# 1, age; 2, race; 3, diabetes mellitus; 4, BMI; 5, NSAID use; 6, education; 7, elevated cholesterol; 8, history of PSA testing; 9, cardiovascular disease; 10, family history of prostate cancer; 11, use of diuretics; 12, use of calcium channel blockers; 13, use of angiotensin-converting enzyme inhibitors; 14, use of angiotensin receptor blockers; 15, use of metformin; 16, use of sulfonylureas; 17, use of insulin; 18, alcohol use; 19, smoking; 20, height; 21, major ancestry; 22, vasectomy; 23, vigorous physical activity; 24, aspirin use; 25, total energy intake; 26, intakes of calcium, fructose, a-linolenic acid, tomato sauce, red meat, fish, supplemental zinc, and high intake of vitamin E; 27, use of other lipid-lowering drugs; 28, use of cardiovascular drugs; 29, use of hormones; 30, prior hospitalisation; 31, chronic disease score; 32, frequency of physician visits; 33, previous neoplasm; 34, use of fibric acids; 35, calendar period of PSA screening; 36, propensity score; 37, cancer; 38, dementia; 39, hypertension; 40, use of 5-α reductase inhibitors; 41, use of α-blockers; 42, follow-up period; 43, study center; 44, interview year; 45, clinical stage and biopsy gleason score; 46, weight; 47, thyroid disease; 48, renal failure; 49, chest pain; 50, mental illness; 51, lung disease; 52, gastro-intestinal disease; 53,number of cores taken; 54, prostate volume; 55, benign prostatic hyperplasia; 56, matching variables.

Further, 11 studies reported RR estimates on the association between long-term statin use and risk of total PCa [5], [8], [10], [14], [17], [25], [28], [31], [34], [37], [38] (**table 2**) and 7 studies presented an examination of statin use in relation to advanced PCa [5], [8], [10], [14], [25], [37], [38] (**table 3**).

**Table 2:** Studies evaluating the association between long-term statin use and risk of total prostate cancer.

Study	RR	95% CI	Total prostate cancer cases	Definition of “long-term” statin use‡
Shannon *et al*., 2005 [17] †	0.22	0.08–0.66	NR	≥2.85 years
Platz *et al*., 2006 [10] *	0.85	0.71–1.03	126	≥5.0 years
Flick *et al*., 2007 [8] *	0.72	0.53–0.99	42	≥5.0 years
Murtola *et al*., 2007 [14] †	1.13	1.00–1.28	1043	≥4 years
Boudreau *et al*., 2008 [25] *	1.06	0.83–1.34	1492	>5 years
Friedman *et al*., 2008 [34] *	1.04	0.93–1.17	NR	>5 years
Agalliu *et al*., 2008 [37] †	1.1	0.7–1.8	45	>10 years
Murtola *et al*., 2010 [5] *	0.70	0.45–1.08	53	≥6.0 years
Coogan *et al*., 2010 [31] †	1.4	0.8–2.5	NR	>10 years
Tan *et al*., 2011 [28] *	0.72	0.53–0.94	42	>5 years
Jacobs *et al*., 2011 [38] *	1.02	0.93–1.12	859	≥5.0 years

RR, Relative risk; CI, Confidence interval; NR, Not reported.

* Cohort studies;

† Case-control studies;

‡ Definition of long-term statin use was taken from original research articles.

**Table 3:** Studies evaluating the association between statin use and risk of advanced prostate cancer.

Study	RR	95% CI	Advanced PCa cases	Definition of “advanced PCa”‡
Platz *et al*., 2006 [10] *	0.51	0.30–0.86	316	Regionally invasive, metastatic, or fatal: stage T3b or worse, N1, M1, or death from PC
Flick *et al*., 2007 [8] *	0.8	0.53–1.19	131	Surveillance, Epidemiology and End Results (SEER) staging system, stage II–IV (regional) or VII (distant)
Murtola *et al*., 2007 [14] †	0.75	0.62–0.91	3,700	Advanced PC; not further defined
Boudreau *et al*., 2008 [25] *	1.22	0.85–1.75	458	Advanced stage cancer defined as regional or distant stage
Agalliu *et al*., 2008 [37] †	0.73	0.48–1.10	181	Advanced PC; not further defined
Murtola *et al*., 2010 [5] *	0.93	0.54–1.58	133	Men with stage T3N0/XM0/X, T4N0/XM0/X, T1-4N1M0 or T1-4N0-1M1 tumors combined
Jacobs *et al*., 2011 [38] *	0.81	0.61–1.08	317	American Joint Committee on cancer stage III or IV, or fatal PC of unknown stage at diagnosis

RR, Relative risk; CI, Confidence interval; PCa, Prostate cancer.

* Cohort studies;

† Case-control studies;

‡ Definition of advanced prostate cancer was taken from original research articles.

### Quality assessment results

With regard to cohort studies, all had an NOS score of 8. In the case-control studies, 11 (92%) were of high quality (NOS score >6), with an average NOS score of 7.7.

### Main analysis

**Figure 2:**
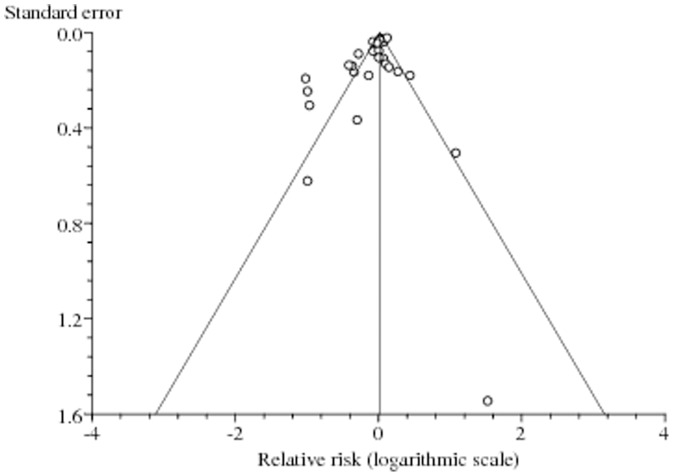
Assessment of publication bias. Funnel plot (publication bias assessment plot) of the relative risk of developing prostate cancer, by the standard error, for all studies. Circles- studies included in the meta-analysis. Relative risks are displayed on a logarithmic scale. p = 0.56 for the Begg's test, and p = 0.12 for the Egger's test.

**Figure 3:**
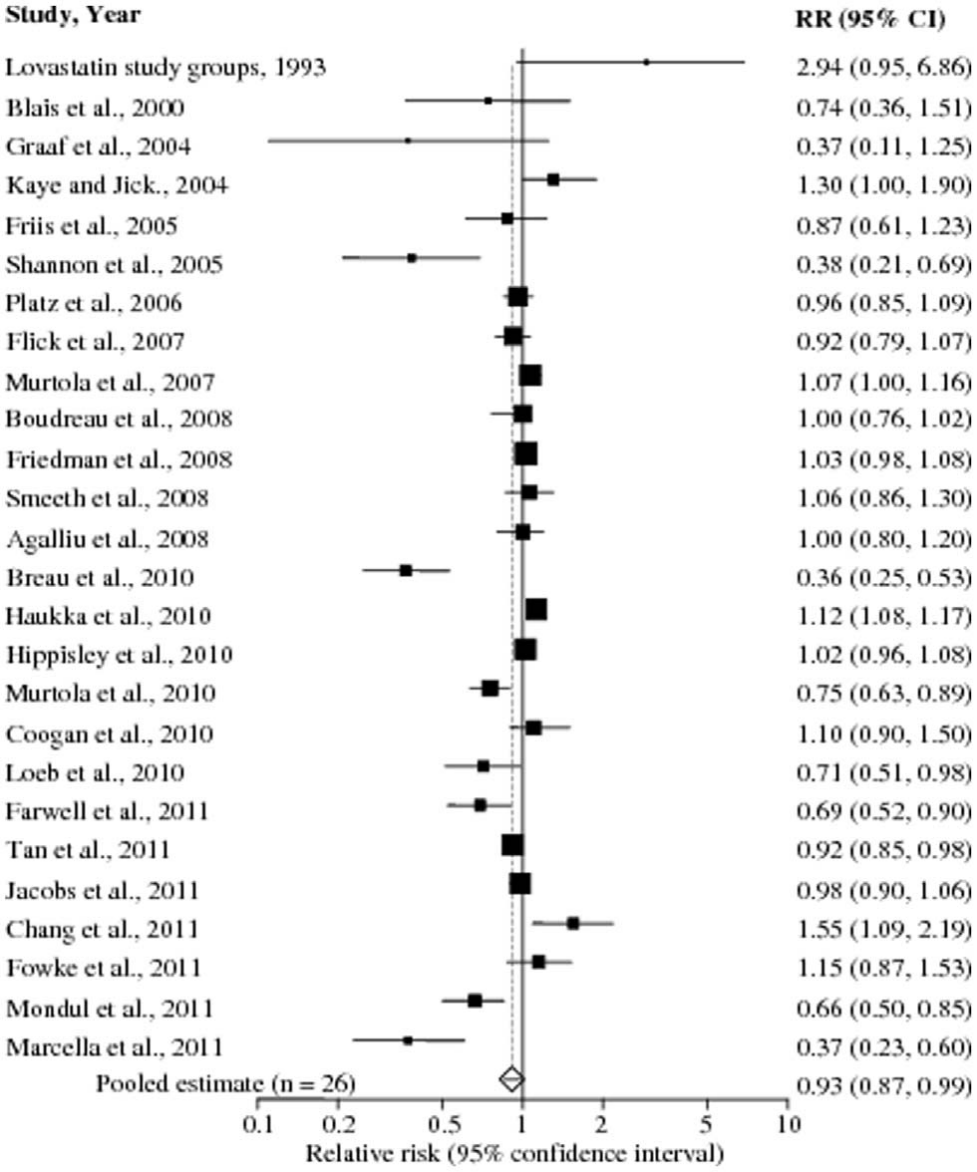
Statin use and risk of prostate cancer. Pooled estimate of relative risk (RR) and 95% confidence intervals (CIs) of total prostate cancer (PCa) associated with statin use based on 27 [in figure study by Sato *et al.* [13] is excluded due to its large CI (RR 4.56, 95% CI 0.06–25.39) and no effect on the final pooled estimated RR] studies (15 cohort and 12 case-control studies) involving more than 1.8 million participants including 56,847 PCa cases. Squares indicate RR in each study. The square size is proportional to the weight of the corresponding study in the meta-analysis; the length of horizontal lines represents the 95% CI. The unshaded diamond indicates the pooled RR and 95% CI (random-effects model).

No publication bias was observed among studies using Begg's p value (p = 0.56), Egger's (p = 0.12) test and the funnel plot, having expected a funnel shape (**figure 2**). Because of significant heterogeneity (p_heterogeneity_<0.001, *I^2^* = 82%), which was to be expected due to some studies showing positive; no; or negative association, a random-effects model was chosen over a fixed-effects model. A pooled analysis of 27 studies found statin use to be associated with significant reduction in the risk of total PCa (RR 0.93, 95% CI 0.87–0.99, p = 0.03). Both multivariable adjusted RR estimates with 95% CIs of each study and pooled RR are shown in **figure 3**.

### Subgroup analyses, sensitivity analysis and cumulative meta-analysis

We found a significant inverse association between statin use and risk of total PCa among cohort studies (RR 0.93, 95% CI 0.87–1.01, p = 0.09) but non-significant inverse association among case-control studies (RR 0.87, 95% CI 0.72–1.05, p = 0.15) (**table 4**). The pooled RR of the studies that were able to either control for PSA levels by comprehensive PSA screening of the entire population or adjusted for PSA testing was 0.91 (95% CI 0.81–1.02, p = 0.13) and for studies which did not adjust for PSA testing the RR was 0.93 (95% CI 0.86–1.01, p = 0.10). Both subgroups presented an inverse association between statin use and PCa. Studies adjusted for BMI and/or ALS showed a significant inverse association (RR 0.88, 95% CI 0.78–0.99, p = 0.04) but this was not observed in studies not adjusted for either BMI and/or ALS (RR 0.96, 95% CI 0.88–1.04, p = 0.35). **A** significant inverse association with studies published after Bonovas *et al*. [24] (RR 0.91, 95% CI 0.84–0.99, p = 0.03) was observed as compared to the studies published in the same time frame included in Bonovas *et al.* (RR 0.95, 95% CI 0.82–1.11, p = 0.58). There was heterogeneity among the studies in these subgroups but no publication bias. Tests for interaction were found non-significant for subgroups of different study design; adjustment for PSA; adjustment for BMI and ALS; and time frame of Bonovas *et al.* analysis (p_interaction = _0.45, 0.76, 0.24 and 0.63, respectively).This confirmed the robustness of the results.

**Table 4:** Overall effect estimates for prostate cancer and statin use according to study characteristics.

	No. of studies	Pooled estimate		Tests of heterogeneity			p_interaction_	Tests of publication bias	
		RR (95% CI)	p-value	*Q* value (d.f.)	p-value	*I^2^* (%)		Begg's p	Egger's p
All studies	27	0.93 (0.87–0.99)	0.03†	145.30 (26)	<0.001	82		0.56∥	0.12
Study design							0.45§		
Cohort	15	0.93 (0.87–1.01)	0.09†	88.60 (14)	<0.001	84		0.56∥	0.07
Case-control	12	0.87 (0.72–1.05)	0.15	56.64 (11)	<0.001	81		0.31∥	0.09
PSA testing							0.76§		
Adjusted	6	0.91 (0.81–1.02)	0.13	14.87 (5)	0.011	66		>0.99∥	0.49
Not adjusted	21	0.93 (0.86–1.01)	0.11	121.23 (20)	<0.001	83		0.53∥	0.04
BMI and ALS							0.24§		
Adjusted	11	0.88 (0.78–0.99)	0.04†	44.23 (10)	<0.001	77		0.54∥	0.22
Not adjusted	16	0.96 (0.88–1.04)	0.37	81.09 (15)	<0.001	81		0.56∥	0.12
Bonovas *et al*. [24] analysis							0.63§		
Before	10	0.95 (0.82–1.11)	0.58	26.62 (9)	0.002	66		0.73∥	0.51
After	17	0.91 (0.84–0.99)	0.03†	118.62 (16)	<0.001	87		0.27∥	0.01
Results for long-term statin use	11	0.94 (0.84–1.05)	0.31	28.80 (10)	0.001	65	0.74§	0.28∥	0.17
Cohort studies	7	0.91 (0.81–1.02)	0.12	14.79 (6)	0.02	59		0.14∥	0.02
Case-control studies	4	0.97 (0.64–1.48)	0.92	9.75 (3)	0.02	69		0.33∥	0.49
Results for advanced prostate cancer	7	0.80 (0.70–0.90)*	<0.001†	8.98 (6)	0.17‡	33	0.25§	0.77∥	0.90
Cohort studies	5	0.85 (0.72–1.00)*	0.04†	7.75 (4)	0.10‡	48		0.81∥	0.62
Case-control studies	2	0.74 (0.62–0.88)*	0.001†	0.01 (1)	0.90‡	-		-	-

PSA, Prostate specific antigen; BMI, Body mass index; ALS, Adverse life style; RR, Relative risk; CI, Confidence interval; d.f., Degree of freedom.

* Relative risk from fixed-effects model due to no heterogeneity among the studies;

† *P* value representing significant inverse association between statin use and prostate cancer;

‡ Statistically significant for homogeneity;

§ Test of interaction was not statistically significant;

∥ Statistically significant for no publication bias.

To test the robustness of our findings, we also carried out a sensitivity analysis. To do this, the overall effect size was calculated by removing one study at a time. This analysis showed no significant variation in pooled RR by excluding two outliers in terms of very low sample size studies: e.g. the Lovastatin study group [11] (RR 0.93, 95% CI 0.86–0.99); and the Sato *et al.* [13] (RR 0.93, 95% CI 0.87–0.99). The same was identified by excluding any of the other studies (RR lay between 0.92–0.96), confirming the stability of present results.

A cumulative meta-analysis of total 27 studies was carried out to evaluate the cumulative effect estimate over time. In 1993, the Lovastatin study groups [11] reported a significant effect estimate of 2.94 (95% CI 0.95–6.86). Between 1993 and 2005 5 studies were published, with a cumulative RR being 0.85 (95% CI 0.53–1.38). Between 2005 and 2011, 21 more publications were added cumulatively, resulting in an overall effect estimate of 0.93 (95% CI 0.87–0.99).

### Results for long-term statin use

**Figure 4:**
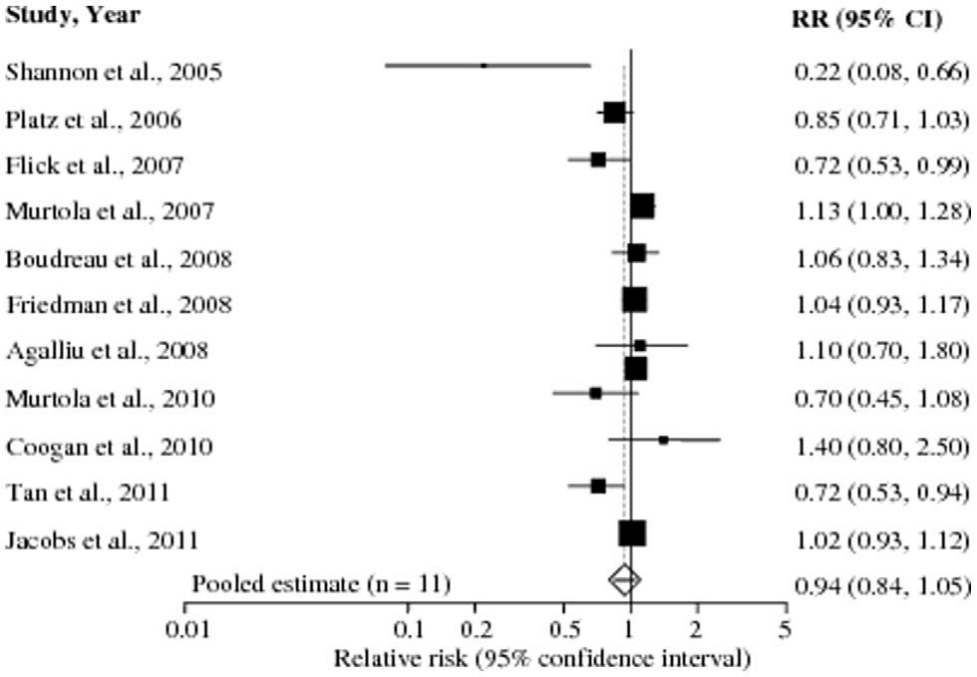
Long-term statin use and risk of prostate cancer. Pooled estimate of relative risk (RR) and 95% confidence intervals (CIs) of total prostate cancer (PCa) associated with long-term statin use based on 11 studies (7 cohort and 4 case-control studies) involving 273,798 participants including 3,702 PCa cases. Squares indicate RR in each study. The square size is proportional to the weight of the corresponding study in the meta-analysis; the length of horizontal lines represents the 95% CI. The unshaded diamond indicates the pooled RR and 95% CI (random-effects model).

Long-term statin use (mostly ≥5 years of use) did not significantly affect the risk of total PCa (RR 0.94, 95% CI 0.84–1.05, p = 0.31). However, there was high evidence of heterogeneity among these studies (p_heterogeneity_ = 0.001, *I^2^* = 65%) but no publication bias [Begg's (p = 0.28), Egger's (p = 0.17)] (**table 4**). Stratification by study design showed both a non-significant inverse association among cohort studies (RR 0.91, 95% CI 0.81–1.02, p = 0.12) and no association among case-control studies (RR 0.97, 95% CI 0.64–1.48, p = 0.92), with the p_interaction_ being calculated at 0.74. The multivariable adjusted RR estimates with 95% CIs of each study and pooled RR are shown in **figure 4**.

### Results for advanced PCa

**Figure 5:**
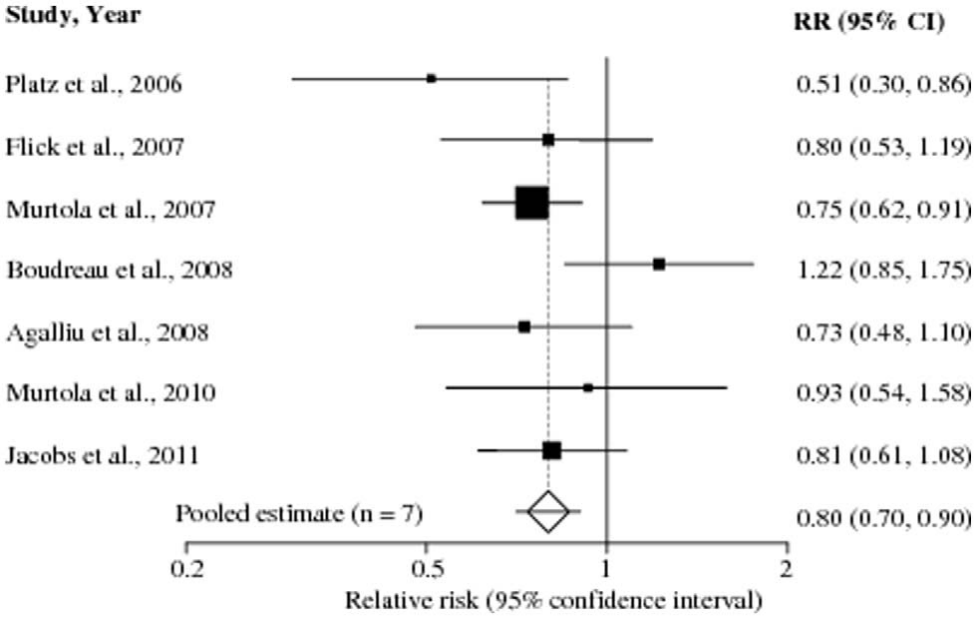
Statin use and risk of advanced prostate cancer. Pooled estimate of relative risk (RR) and 95% confidence intervals (CIs) of advanced prostate cancer (PCa) associated with statin use based on 7 studies (5 cohort and 2 case-control studies) involving 266,209 participants including 5,236 advanced PCa cases. Squares indicate RR in each study. The square size is proportional to the weight of the corresponding study in the meta-analysis; the length of horizontal lines represents the 95% CI. The unshaded diamond indicates the pooled RR and 95% CI (fixed-effects model).

Because of lack of observed heterogeneity among the studies (p_heterogeneity = _0.13, *I^2^* = 38%), a fixed-effects model was chosen over a random-effects model for this analysis. A statistically significant inverse association between statin use and the risk of advanced PCa (RR 0.80, 95% CI 0.70–0.90, p<0.001) was identified. No publication bias was observed among these studies [Begg's (p = 0.90), Egger's (p = 0.54)] (**table 4**). Stratification by study design showed that statistically significant inverse association existed among both the cohort (RR 0.85, 95% CI 0.72–1.00, p = 0.04), and the case-control studies (RR 0.74, 95% CI 0.62–0.88, p = 0.001) with the p_interaction_ being 0.25. The multivariable adjusted RR estimates with 95% CIs of each study and pooled RR are shown in **figure 5**.

## Discussion

In the past decade, the role of statins in the development of PCa has been increasingly understood. With the present updated pooled analysis of 27 observational studies, a 7% reduction in total PCa risk among statin users as compared to non-users was observed and this association remained stable even after the sensitivity analysis.

Present findings regarding total PCa relative risk reduction with statins is at odds with both the Bonovas *et al.* [24] findings and, previous meta-analyses' [21]–[23]. This inconsistency is likely to be associated with the inclusion of 8 new studies published after 2007 that showed a negative association between statin use and risk of total PCa [5]–[7], [25]–[29]. This trend towards significant inverse association from 2007 onwards (publications after Bonovas *et al.* [24] meta-analysis) is clearly demonstrated in the cumulative analysis.

The decreased risk of total PCa in long-term statin users was found here to be non-significant. This is likely to be associated with varying patterns of statin use in different study populations. In many cases, drug use can be irregular, with months of non-use between periods of use [5], [14]. Hence, cumulative amount of statin defined daily doses (DDDs) could be small despite its long duration. Conversely, other studies took into account the use of statins at high doses, which resulted in high cumulative amount of DDDs. From this point of view, it should be noted that the decreasing trend in PCa relative risk has been found to be stronger for cumulative amount of statin use than for duration of its use [5]. Also, the varying definition of “long-term use” could have led to non-significant results. Moreover, the data on long-term use is sparse (only 11 studies with 273,798 participants including 3,702 PCa cases) and divergent (9 out of 11 included studies had shown positive or neutral association), thus neutralizing the effect of statins on PCa risk reduction.

On the other hand, analysis of those reports which specifically examined statin use in relation to the more clinically advanced PCa (*n* = 7) suggested a significant inverse association between them. Although, the staging schemes were likely to be somewhat different, with some studies considering Gleason grade only, whilst others considered both the PCa grade and its stage, most of the studies had considered regionally invasive/metastatic stage III–IV cancer as advanced PCa.

In the subgroup analyses, stratification by study design did not substantially affect the result. Analysis of the subgroup of studies published after Bonovas *et al*., [24] showed significant inverse association while the pooled estimate of the studies covered in Bonovas *et al.* analysis could not demonstrate significant inverse association. This trend of results becomes more discernible with the cumulative meta-analysis showing a change in reporting risk of total PCa from positive in Lovastatin study group [11] to neutral with combined analysis of six studies and then significant inverse association with combined analysis of total 27 studies. This change in the reported association could not be fully explained. The plausible explanation is that low cholesterol might increase cancer risk. This concern has persisted until the early 1990's and it has almost entirely disappeared in the post-statin era [50].

The change can also be because of the change in the screening behavior of the population with regards to PSA testing. FDA has approved serum PSA as a prostate cancer biomarker in 1994, forever changing the diagnostic landscape in the field. With PSA testing, men generally present clinically with early stage disease. Thus, cancer populations considered in studies published prior to 1994 include many more advanced cancers than studies published in the last 10 years. This also suggests that cholesterol levels in the pre-PSA era have a greater chance of being a product of tumor metabolism, leading to a positive; statin use (low cholesterol) -cancer association, whereas cholesterol measures in post-PSA studies are more likely to reflect the cholesterol environment prior to the development of cancer. This would lead to a positive correlation between high cholesterol and prostate cancer risk. Thus, statins showed chemopreventive effect by reducing cholesterol in these patients [50].

There may be a range of different mechanisms behind the apparent reduction of PCa risk in statin users. Specifically, statins inhibit inflammation, angiogenesis, cell proliferation, migration/adhesion, invasion whilst promoting apoptosis, exhibiting selectivity for tumor cells over normal cells [51]. Statins lower the concentration of mevalonate by inhibiting HMG-CoA reductase and thus declining the isoprenylated intermediates that are known to affect signalling pathways along the spectrum from cancer formation to progression [52]. Furthermore, the observed decreased relative risk of PCa among statin users is supported by in-vitro studies [4], [53], [54], which report growth inhibition in prostate-derived cell lines whilst in presence clinically relevant drug concentrations. Apart from the anti-inflammatory and immunomodulatory actions of statins, cholesterol lowering as well as statin pleiotropy through inhibition of the synthesis of isoprenoids has both been implicated in their anticancer properties [55].

The major potential confounding variables in detecting PCa are given by PSA level, BMI and lifestyle factors. Statin use has been shown to affect PSA levels. Recently, a large longitudinal study observed a decline of 4.1% in the median PSA level after initiating a statin [56]. The potential biases introduced by statin influence on PSA and health-seeking behaviour of statin users may be at play but work in opposite directions. Statins lower PSA levels and therefore delay the detection of cancer. Even a small decrease in PSA levels at the population level could translate to lower detection of PCa with an apparent inverse association between cancer risk and statin use. This would lead to a lower risk of total PCa. However, all the diagnosed PCa will progress to advanced disease thus an increased risk of advanced PCa in statin users. On the other hand, statin users are more likely to get PSA testing done [10] and this can be associated with an earlier detection of PCa leading to an increased risk of overall PCa, but a decreased risk of advanced PCa. Lower detection of PCa, among statin users due to decrease in PSA levels (detection bias) can mask the possible protective effect of statins on overall PCa due to a differential use of screening is important for distinguishing the effect of statins from that of screening. An additional subgroup analyses of studies that were able to control for PSA levels by comprehensive PSA screening of the entire population or which adjusted for PSA testing (*n* = 6) [5], [10], [28], [33], [37], [38] was performed. Statin' use remained associated with a reduced overall risk of PCa in both the subgroup of studies, either adjusting or not adjusting for PSA testing.

The possible confounding effect arising due to the indications for which statins are prescribed also needs to be emphasized. Statin users are more likely to be obese or present with ALS behaviour as compared to non-users. This could also affect PCa development or progression. The subgroup analyses of 11 [6], [7], [10], [12], [17], [28], [29], [31], [33], [37], [38] studies which adjusted for BMI and/or ALS revealed a more robust inverse association as compared to those studies which did not adjust for these factors. Obesity and ALS habits such as alcohol, smoking etc. are well known risk factors for the development of PCa [57].

The strength of the present analysis lies in inclusion of 27 observational studies reporting data of more than 1.8 million participants, including 56,847 PCa cases. Our meta-analysis has several limitations. First, we did not search for unpublished studies for original data. Secondly, the included studies were different in terms of study design, confounder adjustments and definitions of drug exposure; long-term statin use; and advanced PCa. The lack of data regarding exposure to PSA testing identified in 21 out of 27 studies included in the present analysis is the most important weakness of the included studies, since PSA testing significantly affects PCa detection [41]. Another, limitation is that only 11 studies have adjusted for potential risk factors like BMI and ALS. Finally, our analysis was restricted to articles in the English language, which may have somewhat biased the results.

In summary, our results suggest a decreased relative risk of PCa in statin users as identified by a combined meta-analysis of 27 observational studies. The results support the hypothesis that cholesterol-lowering with statins is beneficial for both PCa prevention and for clinically important advanced PCa. Further research is needed to address the role of PSA screening and underlying biological mechanisms for this association to confirm the putative protective effects of statins.

## Supporting Information

Checklist S1 **Preferred Reporting Items for Systematic Reviews and Meta-Analyses (PRISMA) checklist.**
(DOC)
